# Laxer Clinical Criteria for Gaming Disorder May Hinder Future Efforts to Devise an Efficient Diagnostic Approach: A Tree-Based Model Study

**DOI:** 10.3390/jcm8101730

**Published:** 2019-10-18

**Authors:** Halley M. Pontes, Bruno Schivinski, Magdalena Brzozowska-Woś, Vasileios Stavropoulos

**Affiliations:** 1 Division of Psychology, School of Medicine, University of Tasmania, Launceston, TAS 7250, Australia; 2 The International Cyberpsychology and Addictions Research Laboratory (iCARL), University of Tasmania, Launceston, TAS 7250, Australia; bruno.schivinski@gmail.com (B.S.); vas@cairnmillar.edu.au (V.S.); 3 RMIT University, School of Media and Communication, 124 La Trobe St, Melbourne, VIC 3000, Australia; 4 Gdansk University of Technology, Department of Marketing, Ul. Narutowicza 11/12, 80-233 Gdansk, Poland; m.brzozowska.wos@gmail.com; 5 Victoria University, School of Psychology, College of Health and Biomedicine, Ballarat Road, Footscray, VIC 3011, Australia

**Keywords:** internet gaming disorder, video games, gaming addiction, problematic gaming, behavioral addictions

## Abstract

Internet Gaming Disorder (IGD) has been recognized in May 2013 and can be evaluated using the criteria developed by American Psychiatric Association (APA). The present study investigated the role each IGD criteria plays in diagnosing disordered gaming. A total of 3377 participants (mean age 20 years, *SD* = 4.3 years) participated in the study. The data collected was scrutinized to detect patterns of IGD using Conditional Inference Tree (Ctree), a sophisticated machine algorithm. Participants provided basic sociodemographic information and completed the Internet Gaming Disorder Scale–Short-Form (IGDS9-SF). The results identified classes of IGD-related symptoms, indicating that endorsing ‘withdrawal’ and ‘loss of control’ increases the probability of disordered gaming by 77.77% while endorsement of ‘withdrawal’, ‘loss of control’ and ‘negative consequences’ increases the probability of disordered gaming by 26.66%. Moreover, lack of endorsement of ‘withdrawal’ and endorsement of ‘preoccupation’ increases the likelihood of disordered gaming by 7.14%. Taken together, the results obtained illustrate that different IGD criteria can present with different clinical weighing as unique diagnostic roles in the development of disordered gaming can be evidenced by each criterion. Moreover, the present findings help inform future revisions of diagnostic manuals and helps enhancing the assessment of IGD in the future. Additional research and clinical implications are discussed.

## 1. Introduction

The concept of addiction has been continuously expanding over the last two decades due to the ongoing developments in the way in which addictive disorders are conceptualized by official medical bodies. Although the definition and operationalization of addiction in itself has not always been entirely consensual due to an inherent diversity in existing theoretical frameworks defining the phenomenon [1], it is currently agreed that addiction is defined beyond substance misuse. This paradigm shift is clearly reflected in the public policy statement from the American Society of Addiction Medicine (ASAM) defining addiction as a primary, chronic disease of brain reward, motivation, memory, and related circuitry, resulting in complex biological, psychological, social, and spiritual negative outcomes and manifestations [2]. Of particular interest in the ASAM’s conceptualization is the fact that addiction can also be reflected when individuals pathologically pursue reward and/or relief by substance use and other behaviors [2]. This expanded notion of addiction has potential far-reaching public health implications given that certain behaviors may result in significant clinical and social impairments due to excessive and pathological engagement with such behavioral activities [3].

In light of the latest conceptual advances within the field of addiction, the American Psychiatric Association (APA) has proposed the construct of behavioral addictions in the fifth revision of the Diagnostic and Statistical Manual for Mental Disorders (DSM-5) [4], and reclassified ‘Gambling Disorder’ as an addictive disorder as opposed to being an impulse control disorder as previously defined in the DSM, further supporting the notion that addiction is now defined beyond mere substance misuse and includes excessive and detrimental engagement in certain behaviors. In addition to these changes, the DSM-5 has introduced for the first time Internet Gaming Disorder (IGD) as a tentative addictive disorder related to excessive video game play warranting further research.

Notwithstanding this, the APA [4] defines IGD as a condition comprising a behavioral pattern marked by persistent and excessive use of video games, resulting in significant clinical impairments or distress during a period of 12 months as indicated by the presence of five out of the nine following diagnostic criteria: (i) preoccupation with games (‘preoccupation’); (ii) withdrawal symptoms when gaming is taken away (‘withdrawal’); (iii) tolerance, resulting in the need to spend increasing amounts of time engaged in games (‘tolerance’); (iv) unsuccessful attempts to control participation in games (‘loss of control’); (v) loss of interest in previous hobbies and entertainment as a result of, and with the exception of, games (‘giving up other activities’); (vi) continued excessive use of games despite knowledge of psychosocial problems (‘continuation’); (vii) deceiving family members, therapists, or others regarding the amount of gaming (‘deception’); (viii) use of games to escape or relieve negative moods (‘escape’); and (ix) jeopardizing or losing a significant relationship, job, or education or career opportunity because of participation in games (‘negative consequences’) [4].

In a similar vein, the World Health Organization (WHO) has recently officialized behavioral addictions in the beta draft revision of the 11^th^ International Classification of Diseases (ICD-11). According to the WHO [5], addictive behaviors are recognizable clinical syndromes associated with significant distress dysfunctions that develop as a result of repetitive rewarding behaviors beyond the use of substance use. Consequently, the WHO now specifies that disorders due to addictive behaviors include Gaming Disorder (GD), which involves both online and/or offline gaming behaviors. With regards to GD, the WHO describes it as a pattern of persistent and recurrent online or offline gaming behaviors occurring within a 12-month time-frame, indicated by: (1) impaired control over gaming (e.g., onset, frequency, intensity, duration, termination, context) (‘loss of control’); (2) increasing priority given to gaming to the extent that it takes precedence over other life interests and daily activities (‘giving up other activities’); and (3) continuation or escalation of gaming despite the occurrence of negative consequences (‘continuation’). Moreover, the behavior pattern is of sufficient severity to result in significant impairment in personal, family, social, educational, occupational or other important areas of functioning (‘negative consequences’) [5].

Interestingly, the operationalization for video game addiction according to the APA and WHO frameworks highlight important discrepancies at the clinical level. Table 1 summarizes and compares the two of the most current diagnostic approaches for video game addiction based on the APA and WHO frameworks. As can be seen from Table 1, it can be argued that the WHO framework for GD takes a laxer approach when defining the phenomenon by reducing the number of clinical criteria necessary to diagnose it, potentially facilitating overdiagnosis and overpathologization. This is a key consideration given the way in which the same psychopathology is diagnosed using a different framework may affect its diagnostic accuracy in relation to its specificity, sensitivity, positive and negative predictive values [6]. The existence of such discrepancies across both diagnostic frameworks makes it paramount to investigate how each diagnostic framework may interfere in relation to the pattern of symptoms underpinning a positive diagnosis. Consequently, the present study will contribute with novel empirical findings that will provide further insights on this particular issue.

Despite the recent debates that have emerged in the literature regarding the social and clinical implications of legitimating behavioral addictions such as video game addiction as an official addictive disorder [7,8,9,10,11,12], it is clear that a large amount of empirical evidence exists supporting the inclusion of video game addiction as a bona fide addiction. According to Pontes [13], there is a fairly significant amount of emerging empirical evidence supporting the validity of video game addiction from the theoretical, empirical, and clinical standpoints. Previous research has also shown that video game addiction shares extensive commonalities with chemical addictions due to substance use and Gambling Disorder in terms of its etiology, phenomenology, neural mechanisms and treatment efficiency [14,15,16,17]. Such commonalities may help explain why video game addiction is often comorbid with different types of substance misuse such as nicotine use disorder [18] and alcohol use disorder [19]. Furthermore, cross-sectional research has shown video game addiction to be systematically linked to a wide range of comorbid disorders, including but not limited to depression [20,21], attention deficit hyperactivity disorder (ADHD) [22,23], obsessive compulsive disorder (OCD) [24,25], and generalized anxiety disorder [26,27]. Although these associations were derived from cross-sectional research, additional longitudinal evidence exists supporting the notion that video game addiction can prospectively contribute to deteriorated overall mental health [28], decreased quality of life [29], reduced levels of sport and exercise engagement [30], poorer academic performance [31,32], and increased levels of depression, anxiety, and social phobias [33].

Although video game addiction has been shown to be both cross-sectionally and longitudinally implicated in several psychological disorders and risky behaviors across all developmental stages [15,34], robust epidemiological studies suggest that only a minority of gamers are affected by this disorder. According to recent findings reported in large-scale representative studies, video game addiction prevalence rates can range anywhere from 0.7% in Norway [35] to 9.3% in Lithuania [36]. More recent epidemiological findings from studies using large and nationally representative samples found comparable prevalence rates according to different geographical regions and age of gamers. More specifically, video game addiction has been found to affect 2% of adults in Macao, China [37] and 1.2% of Norwegian adolescents [38]. Although prevalence rates of video game addiction tend to differ between studies, slightly higher rates are usually reported in Asian countries. Notwithstanding potential cultural differences, other equally plausible reasons underpinning such disparities in prevalence rates of video game addiction may include heterogeneity in the design, assessment method utilized, population investigated, and the diagnostic criteria employed.

Given that video game addiction has been recently recognized as a mental health disorder by the WHO under the nomenclature of GD and that a clear diagnostic framework has been devised by the APA in the DSM-5 for IGD, recent studies have suggested that in order to advance research in this area it is paramount to further investigate the diagnostic validity and feasibility of the existing diagnostic criteria for video game addiction [10,39,40]. This need partially emerged from the fact that there are still significant inconsistencies regarding the potential clinical relevance of certain specific diagnostic criteria for video game addiction within the current IGD diagnostic framework, including but not limited to tolerance and withdrawal symptoms [41,42,43]. Further to the development of the GD clinical criteria by the WHO, investigating the relevance and utility of the nine IGD criteria is indeed key as it can help shape the way in which the phenomenon can be defined and refined in the next revisions of the existing psychiatric diagnostic manuals.

The inconsistencies surrounding the role and clinical relevance of each IGD criteria has been extensively documented by different studies investigating the clinical features of the nine IGD criteria. Accordingly, the first studies to examine the usefulness and validity of the IGD criteria yielded conflicting results. The study by Rehbein, et al. [44] reported that the IGD criteria ‘giving up other activities’, ‘tolerance’, and ‘withdrawal’ were key in the identification of IGD. However, ‘escape’ and ‘preoccupation’ were found to be poor predictors of IGD despite being endorsed at high rates. A follow-up study by Lemmens, et al. [45] found that ‘escape’ did not add significant diagnostic accuracy due to lack of specificity. In terms of poorly performing IGD criteria, Lemmens, Valkenburg and Gentile [45] reported that ‘escape’ presented the lowest specificity levels to distinguish between IGD and non-IGD participants, while Ko, et al. [46] found that ‘deception’ and ‘escape’ presented with the least diagnostic accuracy information when discriminating disordered from non-disordered gamers.

Following this early line of research, several empirical studies adopting Item Response Theory (IRT) emerged in the literature investigating the psychometric characteristics of the nine clinical criteria for IGD providing further important findings regarding the clinical significance and weigh of each IGD criterion [47,48,49,50]. More specifically, Király, Sleczka, Pontes, Urbán, Griffiths and Demetrovics [50] found that ‘continuation’, ‘preoccupation’, ‘negative consequences’, and ‘escape’ were endorsed more frequently in less severe stages of IGD while ‘tolerance’, ‘loss of control’, ‘giving up other activities’, and ‘deception’ were only found in extreme (i.e., severe) clinical cases. Despite the difference in the study design, these findings align well with those reported by Rehbein, Kliem, Baier, Mößle and Petry [44] as ‘giving up other activities’ and ‘tolerance’ were found to help explain that endorsing these criteria associates to a greater probability of a positive IGD diagnosis.

Another recent IRT study conducted by Schivinski, Brzozowska-Woś, Buchanan, Griffiths and Pontes [49] examining the diagnostic properties and measurement performance of the nine IGD criteria on a large sample of gamers found that the criteria ‘continuation’, ‘deception’, and ‘escape’ presented with poor fit in discriminating IGD individuals using the Internet Gaming Disorder Scale–Short-Form (IGDS9-SF) [51], further suggesting that some IGD criteria can have a unique clinical weighing. Moreover, a similar study conducted by Gomez, Stavropoulos, Beard and Pontes [47] on a sample of adolescents from the United States of America using the IGDS9-SF [51] concluded that the criteria ‘giving up other activities’ and ‘withdrawal’ showed greater discrimination properties in comparison to the remaining IGD criteria, further indicating that these two criteria were particularly strong in helping identify gamers with and without exacerbated IGD symptoms.

### The Current Study

Given the rationale discussed above, the present study aims to provide an in-depth empirical examination of the nine IGD criteria as proposed by the DSM-5 [4]. The current study adopts a novel statistical modeling approach to provide much-needed robust data-driven information on the relevance of each IGD criteria and endorsement patterns of these criteria in the context of a potential positive diagnosis of video game addiction. To achieve this goal, this study investigates the main hypothesis that the nine IGD diagnostic criteria will not exhibit the same clinical weigh and predictive diagnostic power in disordered gaming (H1). This hypothesis is grounded on preliminary cross-cultural, cross-sectional, and IRT-based research suggesting that specific IGD criteria may produce differential diagnostic effects and features for diagnosing IGD across different target populations [52,53,54]. The present study contributes to the literature and ongoing discussion on the suitability and clinical validity of IGD criteria by being the first to provide information on the endorsement pathway of the nine IGD criteria in the context of disordered gaming. Finally, the present study also develops a typology of different groups of gamers based on the endorsement patterns of the nine IGD criteria. The development of a symptom-based IGD typology will help explore the process in which researchers and clinicians may be able to distinguish different subgroups of disordered gamers. This is an important step that helps mental health practitioners meaningfully categorize different types of gamers according to their endorsement patterns of IGD symptoms and inform clinical decision-making to enhance therapeutic approaches and clinical outcomes towards treatment and prevention of IGD.

It is envisaged that this study will assist in filling in gaps related to complex clinical aspects surrounding the conceptualization in the APA diagnostic framework regarding IGD as the findings reported will hopefully inform which IGD clinical criteria are mostly relevant when diagnosing IGD. This is likely to reflect and assist in refining the clinical criteria for IGD in future revisions of existing psychiatric diagnostic manuals such as the DSM. At the research level, it is also expected that this study will contribute towards improving the overall quality in the diagnosis of IGD and enhance the consistency of future epidemiological research by providing key information on how the diagnostic criteria for IGD operates at the empirical level and how robust they may be for diagnosing this condition in clinical milieus.

## 2. Method

### 2.1. Participants and Procedures

In the current research, a sample of online gamers was recruited in Poland. Administrators from the three most accessed online gaming forums in Poland (i.e., www.gry-online.pl [more than 970 thousand registered gamers; www.gamesboard.pl [about 50 thousand registered gamers]; and www.gamesfanatic.pl [about 25 thousand registered gamers] were invited to collaborate with the research team to assist in the recruitment process. 

The data collection and participants’ recruitment were conducted by publishing a hyperlink to an online survey held on the service Qualtrics.com. The survey was distributed to gamers registered on the three aforementioned gaming forums alongside their official social media platforms (e.g., Facebook, Twitter, and YouTube). As a characteristic of collecting online data in these circumstances, the exact estimation of how many players had access to the survey hyperlink was not possible to be gauged in the present study. Therefore, the response rate for the study was omitted due to lack of information. Participants were offered no financial compensation to partake in the study. The participation in the study was anonymous. The study was granted approval by the research team’s University Ethics Committee and all stages of the study were in line with the ethical standards of the responsible committee on human experimentation and with the Helsinki Declaration of 1975, as revised in 2005.

In order to be eligible to partake in the study, all participants were asked whether they had played video games in the past 12 months. Responding ‘no’ to this question led to the automatic exclusion of the participants from the study. Therefore, a total of 110 respondents (3.2%) were excluded on this basis, resulting in a final sample of 3377 respondents. Among the remaining eligible participants, the mean age observed was 20 years (*SD* = 4.3, range: 12–49 years). Regarding gender distribution, the sample contained more males 82.67% (*n* = 2789) than females.

### 2.2. Measures

#### 2.2.1. Sociodemographics and Gaming-Related Behaviors

Sociodemographic data included the respondents’ gender, age, and relationship status. Gaming-related behaviors and sociodemographic questions were similar to other studies using the IGDS9-SF [21,27] and included measuring the following three variables: (i) average time spent playing video games from Monday to Friday (weekdays); (ii) average time spent playing video games on Saturday and Sunday (weekends); and (iii) average time spent per gaming session.

The survey controlled for how many years the respondents had been playing video games and if they played from their smart devices. Two additional questions were included asking participants’ agreement with the following statements: ‘I would consider myself addicted to video games’ and ‘I considered myself to be active gamer’.

#### 2.2.2. Internet Gaming Disorder Scale–Short-Form (IGDS9-SF)

The nine-item IGDS9-SF was used to measure IGD [51]. The IGDS9-SF is a short psychometric instrument, which evaluates the nine core criteria defining IGD as defined in the DSM-5 [4]. A summary relating to the operationalization of the nine IGD criteria as measured with the IGDS9-SF and their corresponding clinical criteria is shown on Table 1.

The IGDS9-SF measures the severity of IGD and its detrimental effects by investigating online and offline gaming activities taking place over a 12-month period. The nine items are responded to using a 5-point scale ranging from 1 (‘never’) to 5 (‘very often’). The respondents’ total scores can be obtained by the summation of the responses to the nine items. The total scores can range from 9 to 45 points, where higher scores indicate a higher degree of disordered gaming. To discriminate between disordered and non-disordered gamers, this study implemented Pontes and Griffiths’ [51] suggestion to operationalize endorsement of each diagnostic criteria by recoding responses to each criterion (i.e., IGDS9-SF item) of 5 (‘very often’) as indication of endorsement of the specific criterion. Based on this rationale and recommendation, participants were classed as ‘disordered gamers’ in the present study when endorsing at least five out of nine IGD criteria on the IGDS9-SF, which resulted in a total of 31 (0.96%) potentially disordered gamers as determined by this psychometric diagnostic approach.

This study adopted procedural guidelines used for the cross-cultural adaptation of the Polish version of the IGDS9-SF [55]. Two bilingual translators whose mother tongue was Polish translated the IGDS9-SF from English. Minor discrepancies across the two translations were solved after discussion by the research team members that were fluent in Polish. The Polish IGDS9-SF questionnaire was then back-translated to English by two native English speakers. The two back-translated questionnaires were later compared to the original instrument. The final version of the instrument was then consolidated in a session carried out by the translators and the rest of the research team. The semantic properties of the Polish IGDS9-SF were preserved. 

Lastly, this study assessed face and content validity of the Polish IGDS9-SF by running a pilot study with a sample of 52 video game players in Poland (51% male, mean age = 21.4, *SD* = 3.5). The participants reported no major issues when completing and interpreting each of the questions related to IGD in the Polish IGDS9-SF.

### 2.3. Analytic Strategy and Data Management 

First, the data were checked for accuracy and missing values. To estimate the structure of the missing data, it was performed the Little’s Missing Completely at Random (MCAR) test with the package BaylorEdPsych (R Package for Baylor University Education Psychology Quantitative Course Version 0.5, Baylor University, Walco, TX, United States of America) in R system for statistical computing Version 3.4.1 (https://www.r-project.org). Little’s MCAR test yielded a Chi-Square value of 234.19, DF = 212, *p* = 0.14, therefore, the hypothesis of MCAR was rejected, and the data was deemed to be missing at random. Following this procedure, 155 (4.6%) data points were further eliminated from the analyses for showing missing values on 3 or more items of the IGDS9-SF. The basic descriptive statistics for the excluded subsample are as follows: *n* = 82 female; mean age = 22.6, *SD* = 6.98, range: 14–57 years; and average gameplay session = 2.59 hours, *SD* = 2.54 hours.

The parametric data modeling assumptions were further examined. Thus, to assess for univariate normality, skewness and kurtosis were calculated for the nine items of the IGDS9-SF. The results indicated that no item of the scale yielded an absolute value of skewness above >3.0 and kurtosis >8.0 [56]. Additionally, assessment of univariate outliers involved calculating a standardized composite sum score of the IGDS9-SF items. Respondents were deemed univariate outliers if they scored ± 3.29 standard deviations from the IGDS9-SF *z*-scores. This threshold contains 99.9% of the normally distributed IGDS9-SF *z*-scores [57]. Finally, the critical values based on the chi-square distribution and Mahalanobis distances for each data point were computed to inspect the data for multivariate outliers, leading to no further exclusion of cases. The application of these analytical procedures resulted in a final sample size of 3222 (95.4%) respondents that were used for the later analyses.

### 2.4. Statistical Analyses

The statistical analyses included descriptive analysis of sample structure; reliability analysis of the Polish IGDS9-SF using Cronbach’s alpha and Composite Reliability (CR); construct unidimensionality and criterion-related validity analysis of the IGDS9-SF by estimating a Confirmatory Factor Analysis (CFA) with covariates in a Multiple-Indicator Multiple-Causes model (MIMIC), and estimation of a Conditional Inference Tree (Ctree) model to establish the role of each IGD criterion in the development and diagnosis of IGD. The statistical analyses were conducted using R system for statistical computing Version 3.4.1 with the implementation of the following statistical packages: Psych (Procedures for Psychical, Psychometric, and Personality Research Version 1.8.4, Northwest University, Evanston, UL, United States of America), Lavaan (Latent Variable Analysis Version 0.6-1, Ghent University, Gent, Belgium), and Partykit (A Toolkit for Recursive Partytioning Version 1.2-1, University of Zurich, Zurich, Switzerland).

## 3. Results 

### 3.1. Descriptive Statistics 

The main sociodemographic characteristics of the sample are as follows: in terms of age, 21% (*n* = 677) were aged between 12–16 years, 69.2% (*n* = 2230) were aged between 17–25 years, 8.5% (*n* = 274) were aged between 26–37 years, whereas the remainder (0.6% *n* = 20) were aged between 38–46 years. Furthermore, a total of 70.5% (*n* = 2268) respondents informed not being in a romantic relationship. 

In relation to gaming-related behaviors, the average time spent playing video games was 7.5 hours (*SD* = 6.73 hours) during weekdays (Monday–Friday) and 7.17 hours (*SD* = 5.31 hours) during the weekends (Saturday–Sunday). The average gameplay session was about 2.79 hours (*SD* = 2.12 hours) and about 65.3% of the respondents (*n* = 2203) indicated that they had been playing video games for an average of 8 years (*SD* = 2.9 years); 22.3% (*n* = 717) declared using smart devices to play video games. A large fraction of respondents declared to be active gamers (76.4% *n* = 2462). A total of 14.4% of all respondents (*n* = 464) both ‘agreed’ or ‘strongly agreed’ to the following statement: “I would consider myself addicted to video games”.

Finally, item-related descriptive statistics were as follows: IGDS-SF9 1 ‘preoccupation’ (mean = 2.12, *SD* = 1.13), IGDS9-SF 2 ‘withdrawal’ (mean = 1.84, *SD* = 1.02), IGDS9-SF 3 ’tolerance’ (mean = 2.17, *SD* = 1.14), IGDS9-SF 4 ‘loss of control’ (mean = 1.91, *SD* = 1.06), IGDS9-SF 5 ‘give up other activities’ (mean = 1.71, *SD* = 1.14), IGDS9-SF 6 ‘continuation’ (mean = 2.15, *SD* = 1.28), IGDS9-SF 7 ‘deception’ (mean = 1.89, *SD* = 1.03), IGDS9-SF 8 ’escape’ (mean = 2.88, *SD* = 1.22), and IGDS9-SF 9 ‘negative consequences’ (mean = 1.84, *SD* = 1.06); with a gaming disorder severity of 18.51 (*SD* = 6.42, range: 9–45).

### 3.2. Construct Unidimensionality and Criterion-Related Validity

Construct unidimensionality and criterion-related validity of the Polish IGDS9-SF was evaluated by performing a CFA with covariates in a MIMIC model on its nine items. The MIMIC model was specified so that IGD was predicted by: age, gender, and the total average time spent playing video games during the week (weekdays and weekends). These gaming-related behaviors were chosen based on their predictability of IGD. More specifically, research has demonstrated that IGD is associated with age and gender, with a higher prevalence of IGD being found among younger male players, as well as, greater frequency of gameplay [51,58,59,60,61].

The MIMIC model was computed using the Full Information Maximum Likelihood estimation method (FIML) to account for the missing data. To address violations of normality and normalize the distribution of the sample, the model was specified to yield robust standardized errors by means of bootstrapping. The model was estimated with 5,000 bootstrap samples [62]. 

To inspect the goodness of fit (GOF) of the MIMIC model, established fit indices and thresholds were utilized, thus: χ^2^/d.f. (1; 4); probability level value of the test of close fit (Cfit) > 0.05; Comparative Fit Index (CFI); and Tucker-Lewis Fit Index (TLI) (0.90;0.95); Root Mean Square Error of Approximation (RMSEA) (90% Confidence Interval (CI), 0.05; 0.08); and the Standardized Root Mean Square Residual (SRMR) (0.05;0.08) [63,64,65,66]. Based on this, the MIMIC model yielded the following GOF: χ^2^_(51)_ = 407.48; χ^2^/df = 7.98; CFI = 0.95; TLI = 0.94; RMSEA = 0.04 (90% CI: 0.04–0.05); and SRMR = 0.02. Additionally, all standardized item loadings were above the acceptable threshold of λ_ij_ ≥ 0.50, *p* < 0.001 [67], with the exception of the criterion ‘escape’ (λ_IGDS9-SF 8 ‘escape’_ = 0.47, *p* < 0.001). Although this item presented a poor loading, it was kept it in the model due to its influence on the parameter estimation as (i) it represents a core facet of IGD as defined in the DSM-5 and the fact that (ii) it is often endorsed at high rates of IGD despite its relative weaknesses in predicting IGD and low specificity in discriminating disordered from non-disordered players as shown across several studies [44,45,46,47,50].

**Figure 1:**
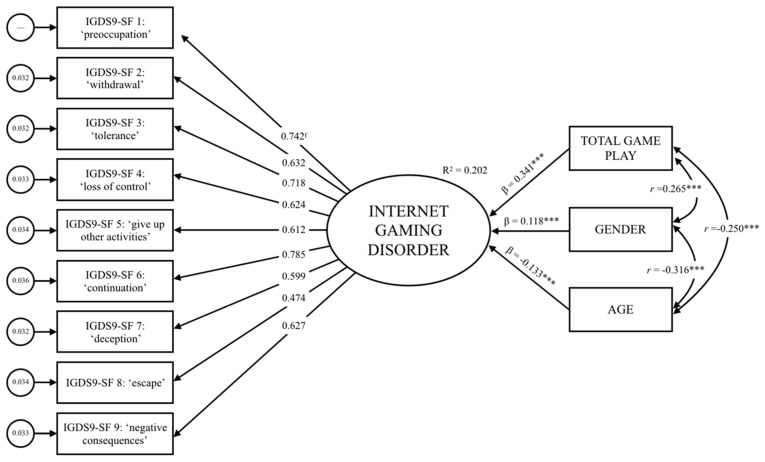
Graphical summary of the validity analysis if the Internet Gaming Disorder (IGD) construct.

As expected, the computations revealed that IGD was impacted by age (β = −0.13, *p* < 0.001), gender (β_ref:female_ = 0.11, *p* < 0.001), and the total average time spent playing video games during weekdays (β = 0.34, *p* < 0.001). Figure 1 lends additional empirical support to the unidimensionality and criterion-related validity of the IGD construct as measured by the IGDS9-SF.

### 3.3. Reliability Analysis

The Cronbach’s alpha for the Polish IGDS9-SF was 0.82 and the CR was 0.86. Both internal consistency coefficients were above the recommended threshold of 0.70 [67,68], further highlighting the effectiveness of the Polish IGDS9-SF to reliably measure IGD-related symptoms. Overall, the internal consistency of the Polish IGDS9-SF was excellent.

### 3.4. Conditional Inference Tree Analysis

In order to directly test H1, the Ctree analysis was performed. This type of analysis is a non-parametric class of regression trees, which implements rule-based procedures with tree-structured regression models. This analysis is capable of handling a large number of exogenous variables, even in the occurrence of multifaceted interactions [69]. The Ctree algorithm assesses the global null hypothesis of independence among the endogenous and exogenous variables implementing a permutation test framework. In the instance of rejecting a hypothesis, the endogenous variable with the highest association to the exogenous variable is chosen and a binary split to this variable is executed. The data is consequently partitioned (split) to smaller homogeneous groups. The algorithm continues to partition the data recursively until the hypothesis is rejected [69]. This analysis prevents selection bias when executing the splitting, therefore, differentiating the Ctree method from other types of analyses exploring homogeneous groups within data such as latent class and cluster analysis. Moreover, Ctree does not split the data according to current patterns in the data (e.g., gaming behavior) as the algorithm returns a set of rules to be fulfilled for an output to happen. In this particular study, the algorithm works to set rules of IGD-related symptoms that increase the likelihood of gaming disorder.

In the literature, Ctree analysis is usually performed to predict several types of behaviors [70] and clinical phenomena such as video game addiction [44,71]. The interpretation of its output is based on nested if–then rules. For instance, if Predictor Y scores ≥4 and Predictor Z scores ≥5 (where scores indicate anything from observable to latent variables), then class (or rule) = 1; if Predictor Y ≥ 4 and Predictor Z < 5, then class = 2; if Predictor Y < 4 then class = 3). Hence, the outcome (i.e., IGD) is later forecasted based on the scores from predictors (i.e., the nine clinical criteria) and their combinations (if any) and the classes generated based on the rules estimated within the analysis.

By estimating the Ctree model in the present sample, a total of five classes (i.e., rules) related to IGD were extracted. The Ctree model was computed with 95% CI whereas the minimum split for partitioning the data was set to 50 cases. The Ctree model was specified using all nine IGD criteria (i.e., IGDS9-SF items) to predict gamers’ IGD diagnostic status (i.e., disordered or non-disordered gaming) controlling for age, gender, and the total average time spent playing video games during the week (weekdays and weekends). To determine the subsample of disordered gamers, a twofold diagnostic approach was taken into account. Firstly, the standard recommendation of criteria endorsement set by the APA in the DSM-5 was adopted [4], and secondly, the recommendation provided by Pontes and Griffiths [51] in the development study of the IGDS9-SF. As a result of this diagnostic approach, disordered gamers were specified in terms of criterion endorsement patterns with high scores, i.e., 5 (‘very often’), for a minimum of five criteria as measured by the IGDS9-SF.

**Figure 2:**
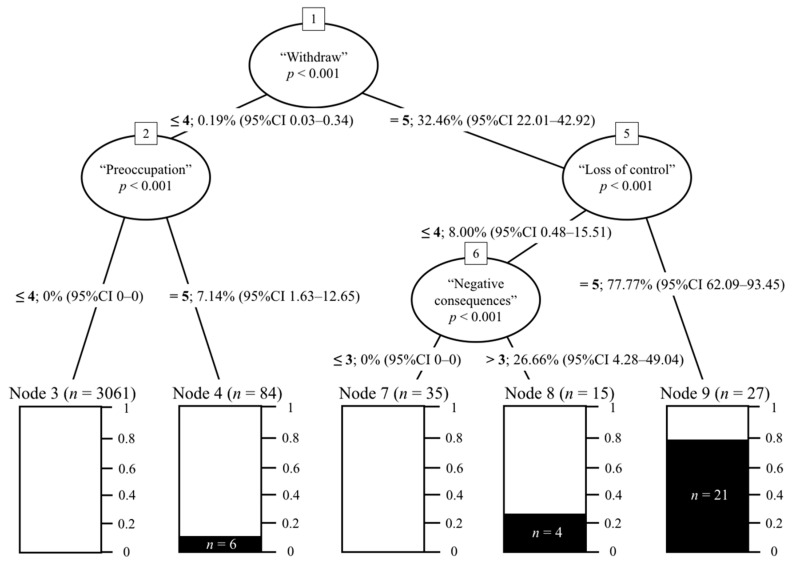
Summary of the Conditional Inference Tree (Ctree) analysis on the nine Internet Gaming Disorder (IGD) criteria across the whole sample (*N* = 3222) and disordered gamers (*n* = 31).

Based on the subsample of gamers meeting the diagnostic criteria for IGD outlined (i.e., *n* = 31), the Ctree analysis revealed that four specific diagnostic criteria from the DSM provided the most diagnostic information and predictive power towards estimating the clinical status of the potentially disordered subsample of gamers. More specifically, ‘withdrawal’, ‘loss of control’, ‘negative consequences’, and ‘preoccupation’ were identified to be key predictors of IGD. The results of the Ctree analysis alongside the rules and patterns identified in the analysis for disordered gamers are summarized in Figure 2 and Table 2.

Overall, the results of the Ctree analysis revealed that among the subsample of disordered gamers, the endorsement pathway of ‘withdrawal’ (i.e., answering with ‘very often’ on item 2) and ‘loss of control’ (i.e., answering with ‘very often’ on item 4) was the case for most disordered gamers (i.e., 67.74%, *n* = 27) as endorsing these two criteria increased the likelihood of IGD by 77.77% (95% CI: 62.09–93.45; *p* < 0.001). This diagnostic pathway underpins Rule 5 (see Table 2) and refers to gamers with ‘Impaired Self-Control’ as they presented with high levels of withdrawal symptoms and deficient self-regulation due to their diminished self-control in relation to gaming. Furthermore, the second most relevant diagnostic pathway among a smaller fraction of disordered gamers (*n* = 4) was related to the following criteria: ‘withdrawal’ (i.e., answering with ‘very often’ on item 2), ‘loss of control’ (i.e., answering with at least ‘very often’ on item 4), and ‘negative consequences’ (i.e., answering with at least ‘sometimes’ on item 9). This second diagnostic pathway increased the likelihood of IGD by 26.66% (95% CI: 4.28–49.04; *p* < 0.001) and denotes Rule 4 which features gamers presenting a ‘Harmful’ gaming pattern due to the experience of withdrawal symptoms, some loss of control, and negative consequences that are typically associated with excessive gaming. Finally, gamers not fully endorsing ‘withdrawal’ (i.e., answering with up to ‘often’ on item 2) but endorsing ‘preoccupation’ (i.e., answering with ‘very often’ on item 1) increased the likelihood of IGD by only 7.14% (95% CI: 1.63–12.65; *p* < 0.001). This diagnostic pathway highlights Rule 2 and features ‘Preoccupied’ gamers that can often experience mild withdrawal symptoms towards gaming due to exacerbated levels of cognitive and behavioral engagement in relation to gaming. 

In addition to the diagnostic pathways and rules reported above, the Ctree analysis provided two additional non-disordered diagnostic pathways underpinning different experiences with gaming. These two diagnostic pathways comprised the vast majority of gamers (*n* = 3096). More specifically, the first non-disordered diagnostic pathway included gamers that presented low levels of ‘withdrawal’ (i.e., answering with up to ‘often’ on item 2) and ‘preoccupation’ (i.e., answering with up to ‘often’ on item 1). These gamers made up for the majority of the gamers recruited (i.e., 95%, *n* = 3061) and underpins Rule 1, which features a ‘Healthy’ gaming pattern due to the lack of increased symptomatology and impairments stemming from excessive gaming. Finally, another non-disordered diagnostic pathway featuring a minority of gamers also emerged (i.e., 1.09%, *n* = 35). This diagnostic pathway included gamers endorsing ‘withdrawal’ (i.e., answering with ‘very often’ on item 2) but presenting low levels of ‘loss of control’ (i.e., answering with up to ‘often’ on item 4) and ‘negative consequences’ (i.e., answering with up to ‘sometimes’ on item 9). Furthermore, this diagnostic pathway describes Rule 3, which features gamers with a ‘Low Risk’ profile for developing IGD as they appear to engage in a gaming style that may include occasional excessive gaming to avoid the experience of unpleasant feelings that does not totally compromise their self-control, which would then lead to the experience of greater symptom severity of IGD alongside its accompanying detrimental consequences. Taken together, the results of the Ctree provide empirical support to H1 as the nine IGD diagnostic criteria exhibited different clinical weigh and predictive diagnostic power among disordered gamers.

## 4. Discussion

Based on a decision tree model and the preliminary evidence reviewed, the present study sought to investigate how the nine IGD diagnostic criteria perform in a large and heterogenous sample of gamers. More specifically, the study hypothesized that the IGD criteria would exhibit differential effects regarding their clinical weighting and predictive diagnostic power in disordered gaming.

To achieve this goal and shed light on the main hypothesis under investigation, the psychometric properties of the Polish IGDS9-SF [51] were scrutinized to ascertain the suitability of this psychometric tool in the assessment of IGD and to ensure whether it is psychometrically fit for this purpose in the current sample. More specifically, the study examined its reliability, construct and criterion-related validity by computing a MIMIC model using structural equation modeling (SEM) to estimate a measurement model for IGD based on the nine diagnostic criteria from the APA as measured by the IGDS9-SF. The model also accounted for potential effects stemming from observable variables such as time spent playing, gender, and age as the literature suggests that these variables are often relevant predictors of IGD [22,58,72,73]. Overall, based on the results obtained from these analyses it was possible to conclude that the Polish IGDS9-SF presented with excellent psychometric properties concerning its validity and reliability to assess symptoms and the severity of IGD in the sample recruited. The findings encountered were aligned with those of previous studies on IGD using the IGDS9-SF reporting a high reliability, consistent validity indicators and unidimensionality across different samples and study designs [21,52,54,74,75,76,77]. Further to this, the study estimated a tree-based model using the nine IGD criteria to investigate H1 (i.e., the nine IGD diagnostic criteria will not exhibit the same clinical weigh and predictive diagnostic power in disordered gaming).

Although previous research on IGD has relied on tree-based models to further explore the phenomenon [44], the present study adopted a complex nested if–then set of rules to estimate a tree-based model that allowed operationalizing each IGD criteria as continuous variables as opposed to binary variables as previous research did [44]. Based on the extant literature, it can be argued that operationalizing the diagnostic criteria for IGD in a binary way takes away the potential to explore the complexity of each symptom by unrealistically assuming their endorsement or non-endorsement and denying the fact that they can be endorsed within a continuum of symptom severity as with most psychological disorders. This, however, is rarely the case as it does not account for the clinical intricacies involved in the phenomenology, clinical course, and diagnostic approach of IGD as it has been extensively reported by recent case studies of disordered gamers [78,79]. Additionally, adopting a severity-based approach supports the well-established notion that IGD symptoms occur within a spectrum of problem-severity whereby some criteria that may be endorsed at higher severity levels may not be similarly endorsed at lower severity levels [47,48,50,80].

Nevertheless, the results of the Ctree analysis revealed that out of the nine IGD criteria, ‘withdrawal’, ‘preoccupation’, ‘loss of control’, and ‘negative consequences’ emerged as the most relevant diagnostic criteria, further suggesting five distinct endorsement pathways leading to the identification of different subgroup of gamers according to the endorsement patterns of each criteria. More specifically, a subgroup of disordered gamers with exacerbated ‘Impaired Self-Control’ was identified. Accordingly, gamers within this subgroup comprised the vast majority of all potentially disordered gamers in the sample. These gamers exhibited a clear endorsement pathway comprising ‘withdrawal’ and ‘loss of control’, which led to a significant increase in the likelihood of a positive IGD diagnosis by the majority of the likely disordered gamers in the sample. This finding can be framed within the large body of research supporting IGD as a bona fide addictive disorder and its perilous effects on self-control and self-regulation. From the structural characteristics (i.e., how video games are developed) standpoint, it is known that certain structural characteristics present with greater addictive risk for gamers, such as those games embedding a refined and complex in-game intermittent reward schedule, further facilitating loss of control over game use [81,82]. From a neurobiological standpoint, loss of control and deficient self-regulation within disordered gaming is partially supported by emerging neurobiological research suggesting that various aspects of cognitive control (i.e., inhibitory control, error processing, attentional control) appear to be implicated in IGD as decreased inhibitory control coupled with increased impulsivity levels may constitute a neurocognitive risk factor in disordered gamers [83].

Additionally, a second subgroup of gamers presenting a ‘Harmful’ gaming pattern emerged in the Ctree analysis. This group included a very small proportion of potentially disordered gamers from the sample. Although these gamers still experienced augmented levels of ‘withdrawal’ and ‘loss of control’, they have also presented with some degree of ‘negative consequences’. These gamers were labelled as presenting a ‘Harmful’ gaming pattern due to the potential detrimental effects experienced as a result of the accompanying ‘negative consequences’ of their gaming behavior. Notwithstanding this, the likelihood of these gamers presenting a positive IGD diagnosis was only about 26.66%, which is significantly less than gamers with ‘Impaired Self-Control’. Thus, ‘Harmful’ gaming marked by the experience of less severe ‘negative consequences’ stemming from excessive gaming may be an important risk factor in subsequent development of IGD due to the way in which daily activities can be disrupted by the gaming behavior. In this context, based on the findings encountered in the present study, harmful gaming can be defined as a behavioral gaming pattern encompassing the experience of withdrawal symptoms and loss of control to some extent, further leading to occasional experience of negative outcomes due to excessive gaming. This definition substantiates existing conceptualizations of harmful use of technology defining this phenomenon as uncontrolled preoccupations or pleasure-seeking activities marked by increased tolerance and withdrawal symptoms despite the impairment and distress associated with it’ [84].

Moreover, the Ctree analysis revealed a subgroup of ‘Preoccupied’ gamers that often experience less severe ‘withdrawal’ despite the increased levels of ‘preoccupation’ towards gaming. This diagnostic pathway included a reduced portion of gamers and was not as problematic because it represented a small likelihood of 7.14% of a positive IGD diagnosis. Although this subgroup presented with a relatively low probability of a positive diagnosis, it is clear that this has resulted from the fact that the IGD criteria and their endorsement patterns within this subgroup combined elements of what has been defined as ‘core criteria’ and ‘peripheral criteria’ related to IGD. More specifically, the seminal works by Charlton and Danforth [85,86] proposed that not all diagnostic criteria for IGD present with the same relevance, leading Charlton and Danforth [85] to suggest that ‘core criteria’ are central when defining IGD as they include the experience of conflicts, withdrawal symptoms, relapse, and behavioral salience. Moreover, ‘peripheral criteria’ are not so central in the diagnosis of IGD due to their non-pathological nature, including criteria related to the experience of cognitive salience/preoccupation, tolerance, and euphoria [85]. Although several follow-up studies were able to corroborate and support the role of both ‘core criteria’ and ‘peripheral criteria’ in relation to IGD [35,58,72,87], the present findings in relation to the subgroup of ‘Preoccupied’ gamers seem justified by the experience of both core and peripheral symptoms of IGD.

Finally, as expected, the Ctree analysis revealed that the majority of the gamers recruited either fell under the subgroup of ‘Healthy’ or ‘Low Risk’ gamers due to the type of diagnostic endorsement pathway present in these gamers. For these gamers, the experience of detrimental and several impairments stemming from excessive gaming was not a dominant theme. This further corroborates the idea that in general, video game playing is a healthy and desirable activity boasting a wide range of beneficial effects to the majority of gamers [88,89].

Although the present findings are robust and sound, they are not without potential limitations emerging from the chosen study design and sampling strategy used in the present study. Moreover, caution is needed when interpreting the findings reported in this study as they may not fully reflect the actual clinical reality of disordered gamers as the sample recruited to the present study was a community-based and non-probability sample of normative gamers. Additionally, the study relied on a cross-sectional design which naturally hinders any viability for causal interpretations of the results presented.

## 5. Conclusions: Implications for Future Research and Diagnostic Practices

Despite the fact that the present research provided further insights on the role of each IGD criteria in terms of their diagnostic properties. This study paves the way to future research examining the effectiveness of existing diagnostic approaches to IGD. Moreover, the present findings warrant subsequent in-depth scrutiny of the nine IGD criteria within clinically diagnosed samples using a gold standard. Consequently, the authors of the present study encourage researchers conducting similar studies to obtain a clinical gold standard when diagnosing IGD to ensure the diagnosis goes beyond the realm of psychometric testing. A potential fruitful way to achieve this step may be through semi-structured interviews of gamers within a clinical setting by a trained psychiatrist using the nine IGD criteria from the DSM-5. Moreover, conducting research on IGD using latent profile analysis to understand the clinical aspects of this condition within latent profiles of homogenous groups would also help informing the basic etiological features of IGD, which can be a valuable insight for psychologically-driven treatment approaches.

In addition to research-related implications, the present study has the potential to inform key stakeholders involved in the revision of the existing psychiatric diagnostic manuals by providing clear data-driven insights on how each of the nine IGD criteria can contribute to a potential positive diagnosis. The findings presented here can be reliably used to aid refinement of the existing IGD criteria by means of an evidence-based approach, which is in line with previous recommendations that researchers should gain greater knowledge regarding how the nine IGD criteria operate both in the general population and clinical levels [39,90,91].

In addition to potentially contributing to refining the existing IGD diagnostic framework, the present findings are timely as they inform potential underlying issues with the proposed draft version of the GD diagnostic criteria by the WHO. This study has direct implications to how video game addiction is currently operationalized under the new framework proposed by the WHO as the evidence obtained suggests that ‘withdrawal’ plays a key role in predicting greater and low likelihood of video game addiction. This finding is also congruent with previous empirical research suggesting that withdrawal symptoms are a core feature of video game addiction [85]. Given that the WHO does not acknowledge the experience of withdrawal symptoms as being a core symptom of GD, it could be argued that the diagnostic criteria for GD is laxer in comparison to the nine IGD criteria which presents a greater number of clinical criteria, potentially making it harder for someone to meet all the conditions to be clinically diagnosed. Although the present research cannot provide concrete answers towards this issue, it is worth considering how the newly developed GD diagnostic framework by the WHO may contribute towards inflated prevalence rates of video game addiction due to using fewer (i.e., more relaxed) diagnostic criteria to diagnose video game addiction. To this end, the present authors call to a critical and empirically-driven reflection on how the promising GD diagnostic framework should be defined in order to acoid overpathologization of normal behaviors [92].

## Figures and Tables

**Table 1:** The operationalization of video game addiction according to the nine Internet Gaming Disorder (IGD) as measured with the Internet Gaming Disorder Scale–Short-Form (IGDS9-SF), their corresponding clinical criteria and comparison against the criteria for gaming disorder within the 11th International Classification of Diseases (ICD-11).

Item	IGDS9-SF Item Wording	Clinical Criteria	Included in the ICD-11?
1	Do you feel preoccupied with your gaming behavior? (Some examples: Do you think about previous gaming activity or anticipate the next gaming session? Do you think gaming has become the dominant activity in your daily life?)	Preoccupation	No
2	Do you feel more irritability, anxiety or even sadness when you try to either reduce or stop your gaming activity?	Withdrawal	No
3	Do you feel the need to spend increasing amount of time engaged gaming in order to achieve satisfaction or pleasure?	Tolerance	No
4	Do you systematically fail when trying to control or cease your gaming activity?	Loss of control	Yes
5	Have you lost interests in previous hobbies and other entertainment activities as a result of your engagement with the game?	Giving up other activities	Yes
6	Have you continued your gaming activity despite knowing it was causing problems between you and other people?	Continuation	Yes
7	Have you deceived any of your family members, therapists or others because the amount of your gaming activity?	Deception	No
8	Do you play in order to temporarily escape or relieve a negative mood (e.g., helplessness, guilt, anxiety)?	Escape	No
9	Have you jeopardized or lost an important relationship, job or an educational or career opportunity because of your gaming activity?	Negative consequences	Yes

**Table 2:** Summary of the Conditional Inference Tree-derived rules alongside each Internet Gaming Disorder (IGD) criteria endorsement pathway according to each subtype of gamer within the sample (*N* = 3377).

Rule	IGD Criteria Endorsement Pathways			Gamer Subtype
1	‘withdrawal’ ≤ 4; 0.19% (95% CI 0.03–0.34)	‘preoccupation’ ≤ 4; 0% (95% CI 0–0), *n* = 3061		‘Healthy’
2	‘withdrawal’ ≤ 4; 0.19% (95% CI 0.03–0.34)	‘preoccupation’ = 5; 7.14% (95% CI 1.63–12.65), *n* = 84		‘Preoccupied’
3	‘withdrawal’ = 5; 32.46% (95% CI 22.01–42.92)	‘loss of control’ ≤ 4; 8.00% (95% CI 0.48–15.51)	‘negative consequences’ ≤ 3; 0% (95% CI 0–0), *n* = 35	‘Low Risk’
4	‘withdrawal’ = 5; 32.46% (95% CI 22.01–42.92)	‘loss of control’ ≤ 4; 8.00% (95% CI 0.48–15.51)	‘negative consequences’ > 3; 26.66% (95% CI 4.28–49.04), *n* = 15	‘Harmful’
5	‘withdrawal’ = 5; 32.46% (95% CI 22.01–42.92)	‘loss of control’ = 5; 77.77% (95% CI 62.09–93.45), *n* = 27		‘Impaired Self-Control’

Note: Answers given by participants to each IGD criterion as measured with the Internet Gaming Disorder Scale–Short-Form (IGDS9-SF) included 1 = ‘Never’, 2 = ‘Rarely’, 3 = ‘Sometimes’, 4 = ‘Often’, and 5 = ‘Very Often’. Endorsement of an IGD criterion was operationalized with answers equal to 5 = ‘Very Often’.
